# Supplementary figures and images for: Cell-based and multi-omics profiling reveals dynamic metabolic repurposing of mitochondria to drive developmental progression of Trypanosoma brucei
Source: PLoS Biol. 2020 Jun 10;18(6):e3000741. doi: 10.1371/journal.pbio.3000741 (PMC7307792; doi:10.1371/journal.pbio.3000741)

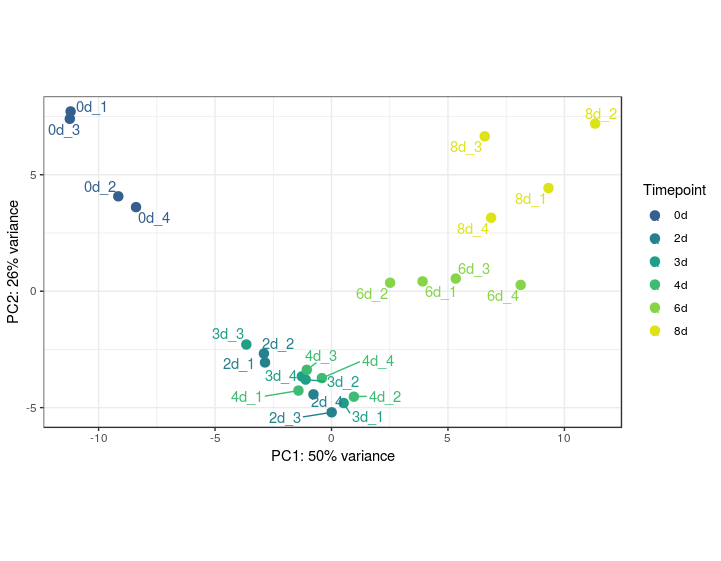

Supplement: S1 Fig — PCA, principal component analysis; RBP6, RNA binding protein 6. (TIF) [file pbio.3000741.s001.tif]

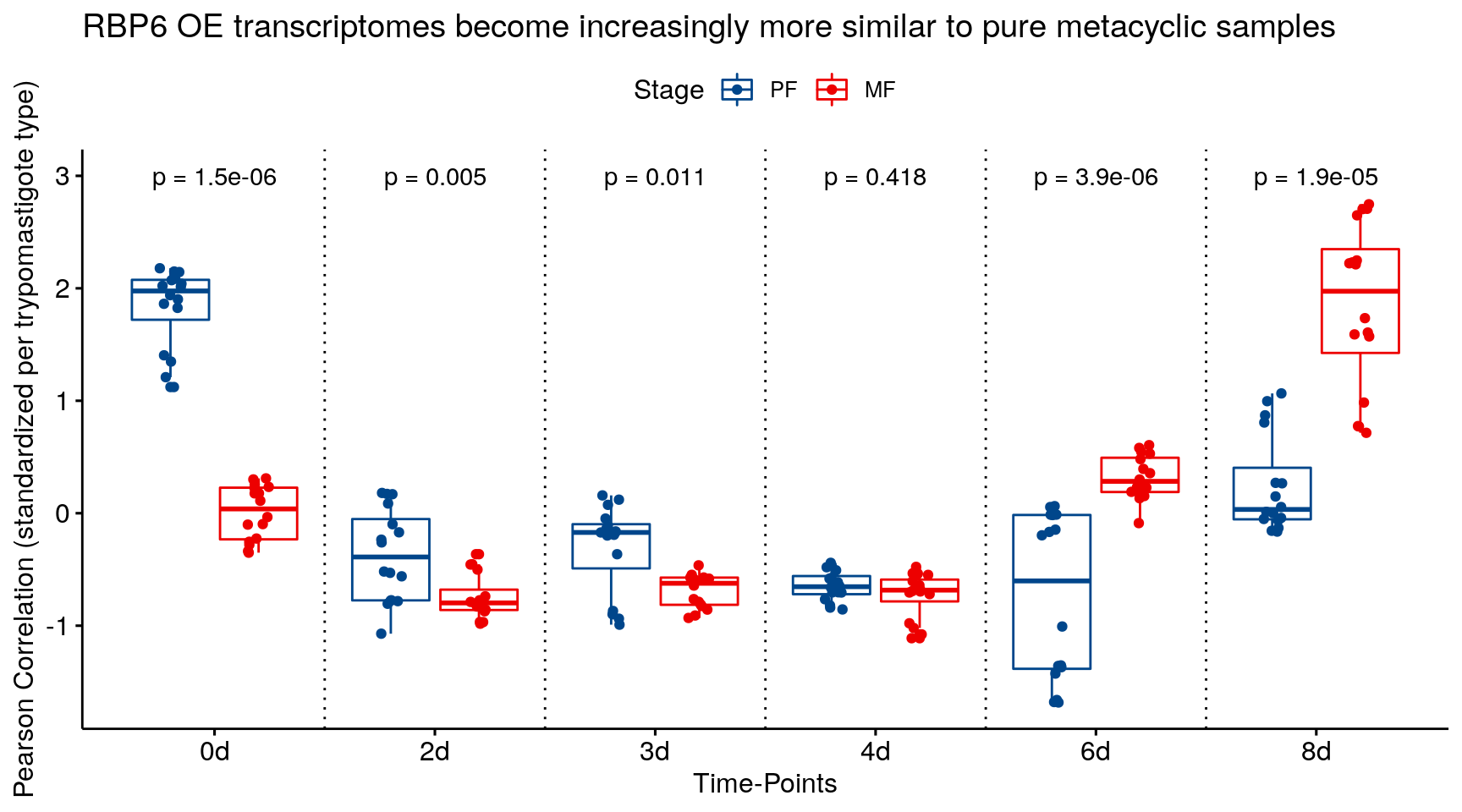

Supplement: S3 Fig — The Pearson correlation values reflect the similarity of the transcriptomes of the respective time points to the two trypomastigote types—procyclic and metacyclic forms. RBP6, RNA binding protein 6. (PNG) [file pbio.3000741.s003.png]

Color Key

and Density Plot

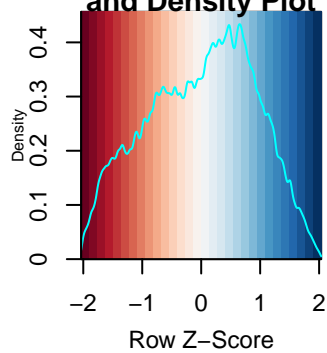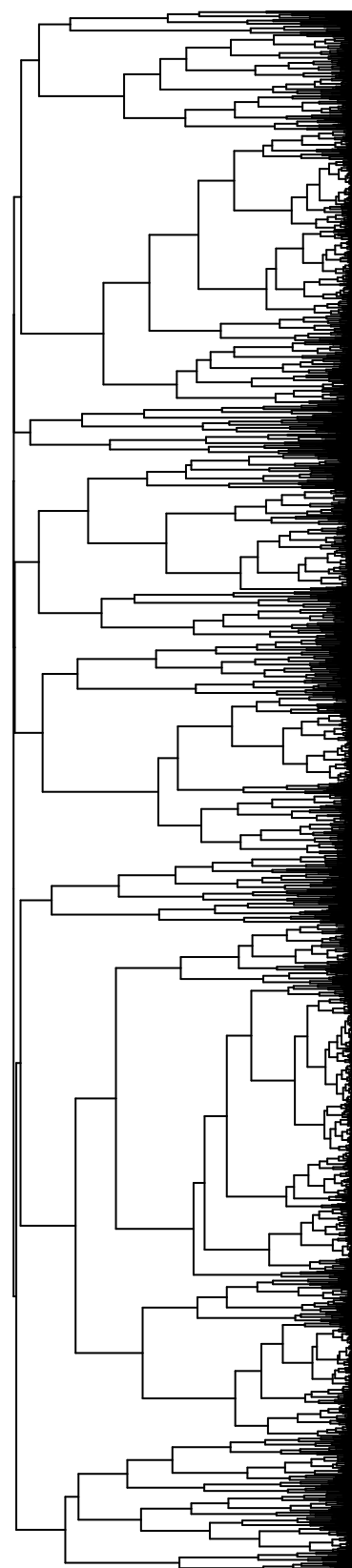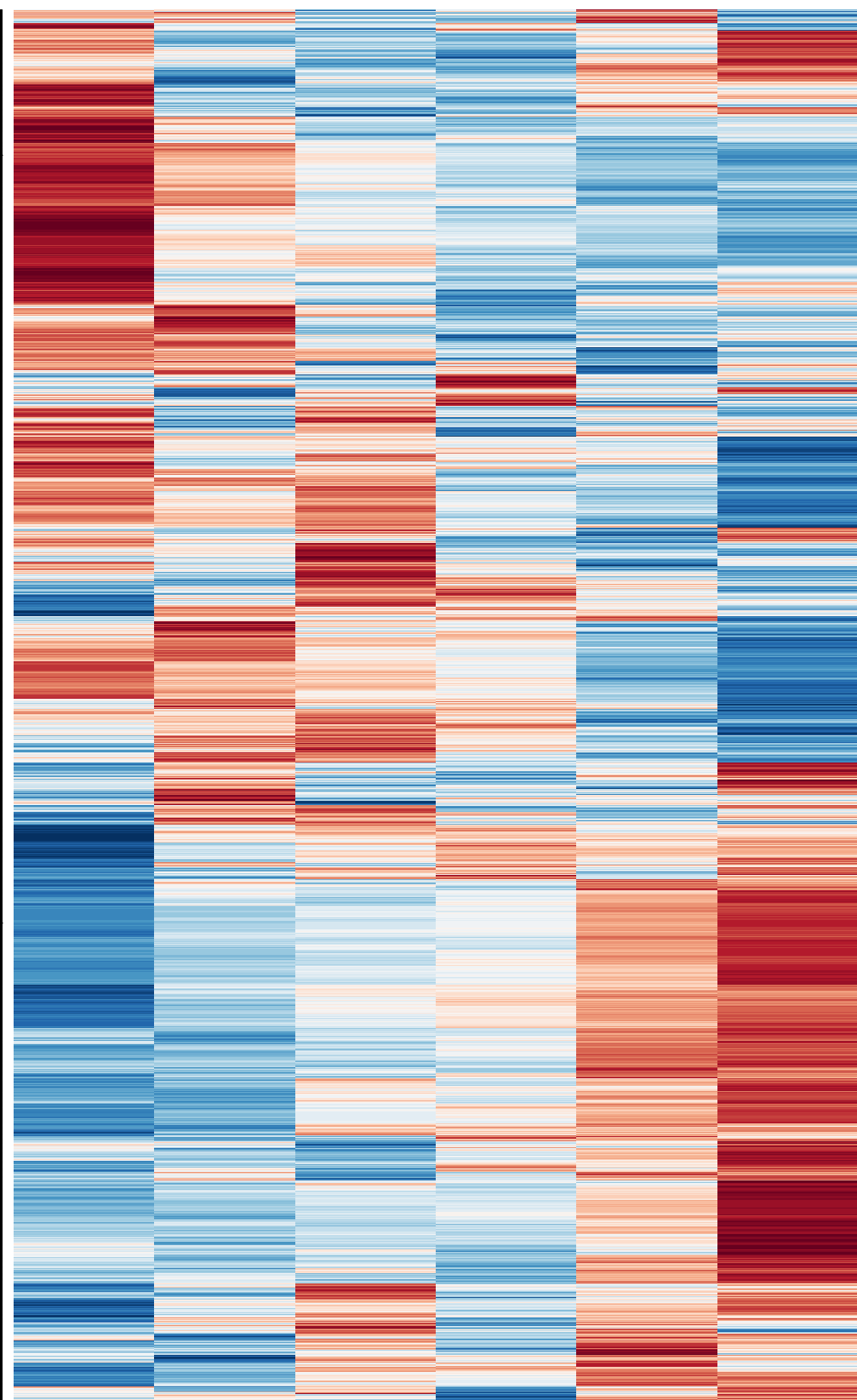

0d

2d

3d

4d

6d

8d

Supplement: S4 Fig — The heatmap encompassing 5,227 z-scored LFQ quantified protein groups illustrates significant proteome remodeling during RBP6-induced differentiation. LFQ, label-free quantification; RBP6, RNA binding protein 6. (PDF) [file pbio.3000741.s004.pdf]

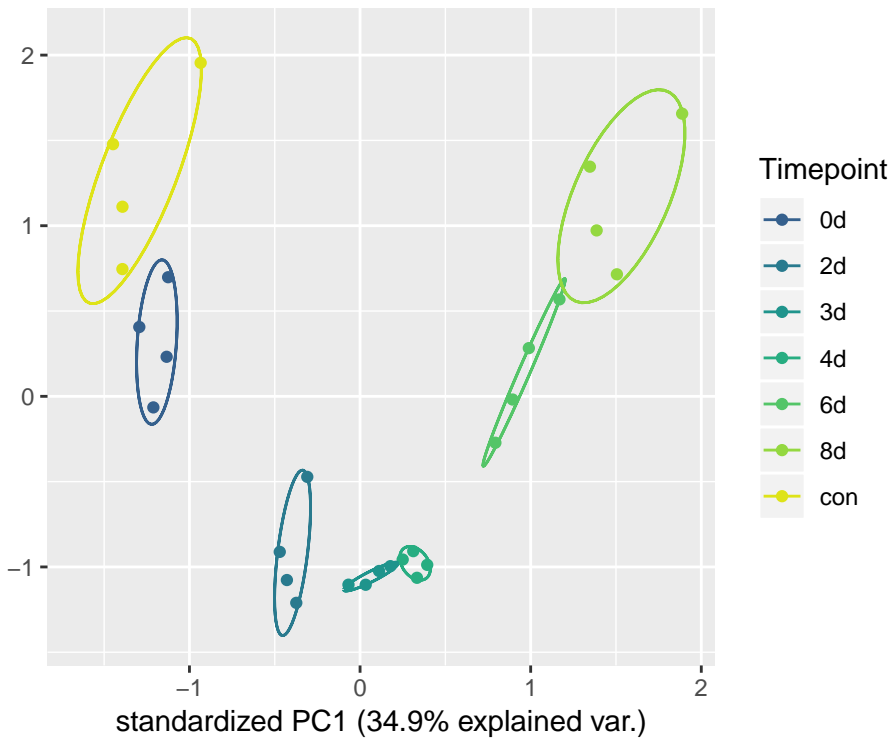

Supplement: S5 Fig — PCA of the proteomic samples shows the reproducibility of replicates. PCA, principal component analysis. (PDF) [file pbio.3000741.s005.pdf]

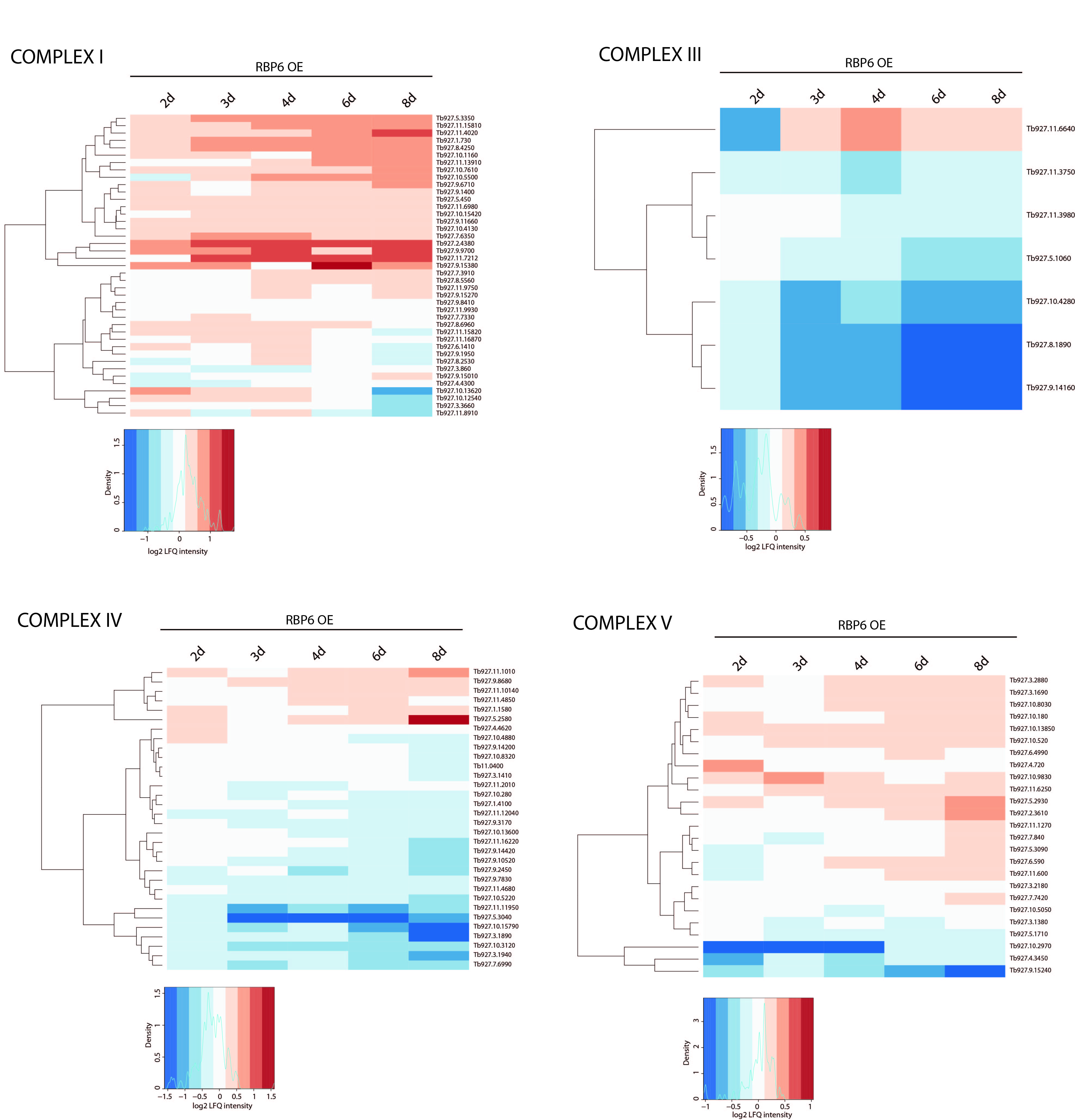

Supplement: S6 Fig — The color key differs for each map and is always located below the heatmap. LFQ, label-free quantification; RBP6, RNA binding protein 6. (JPG) [file pbio.3000741.s006.jpg]

**RBP6 OE NON**

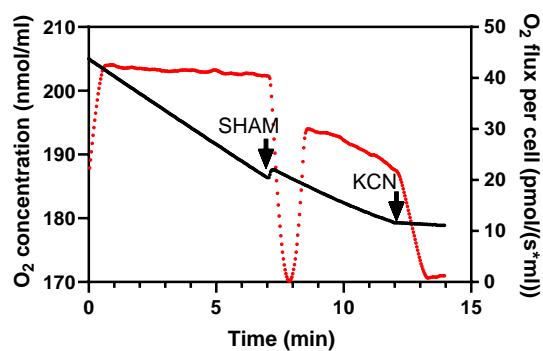

**RBP6 OE IND2**

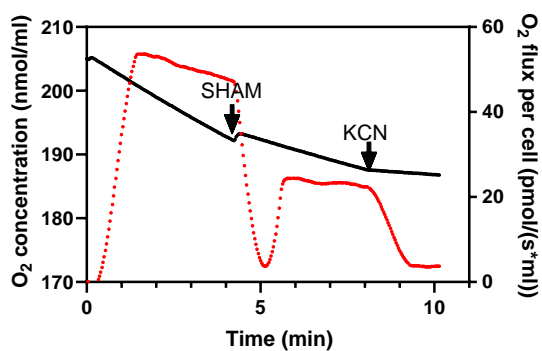

**RBP6 OE IND 4**

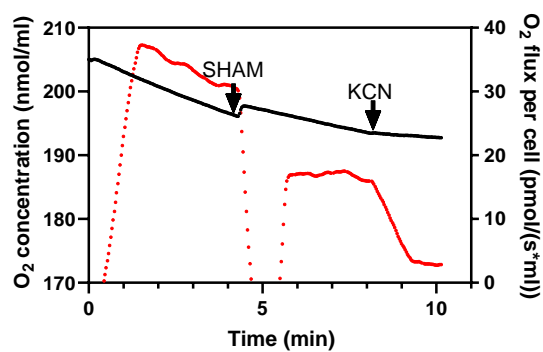

**RBP6 OE IND 6**

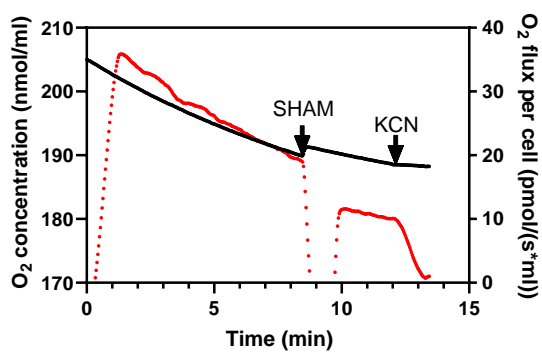

Supplement: S7 Fig — The black lines show a decreasing concentration of oxygen in the buffer (left y-axis), while the red line shows O2 flux per cell (right y-axis). Inhibition of AOX-mediated respiration was induced by addition of SHAM. The addition of KCN inhibited respiration via complex IV. AOX, alternative oxidase; KCN, potassium cyanide; RBP6, RNA binding protein 6; SHAM, salicylhydroxamic acid. (PDF) [file pbio.3000741.s007.pdf]
