# Supplementary material for: Cell-based and multi-omics profiling reveals dynamic metabolic repurposing of mitochondria to drive developmental progression of Trypanosoma brucei
Source: PLoS Biol. 2020 Jun 10;18(6):e3000741. doi: 10.1371/journal.pbio.3000741 (PMC7307792; doi:10.1371/journal.pbio.3000741)
Supplement: S2 Fig — The genes with fold change larger than 2 or smaller than 0.5 (with Benjamini-Hochberg–corrected P values smaller than 0.05) are highlighted in red. RBP6, RNA binding protein 6. (PDF) [file pbio.3000741.s002.pdf]

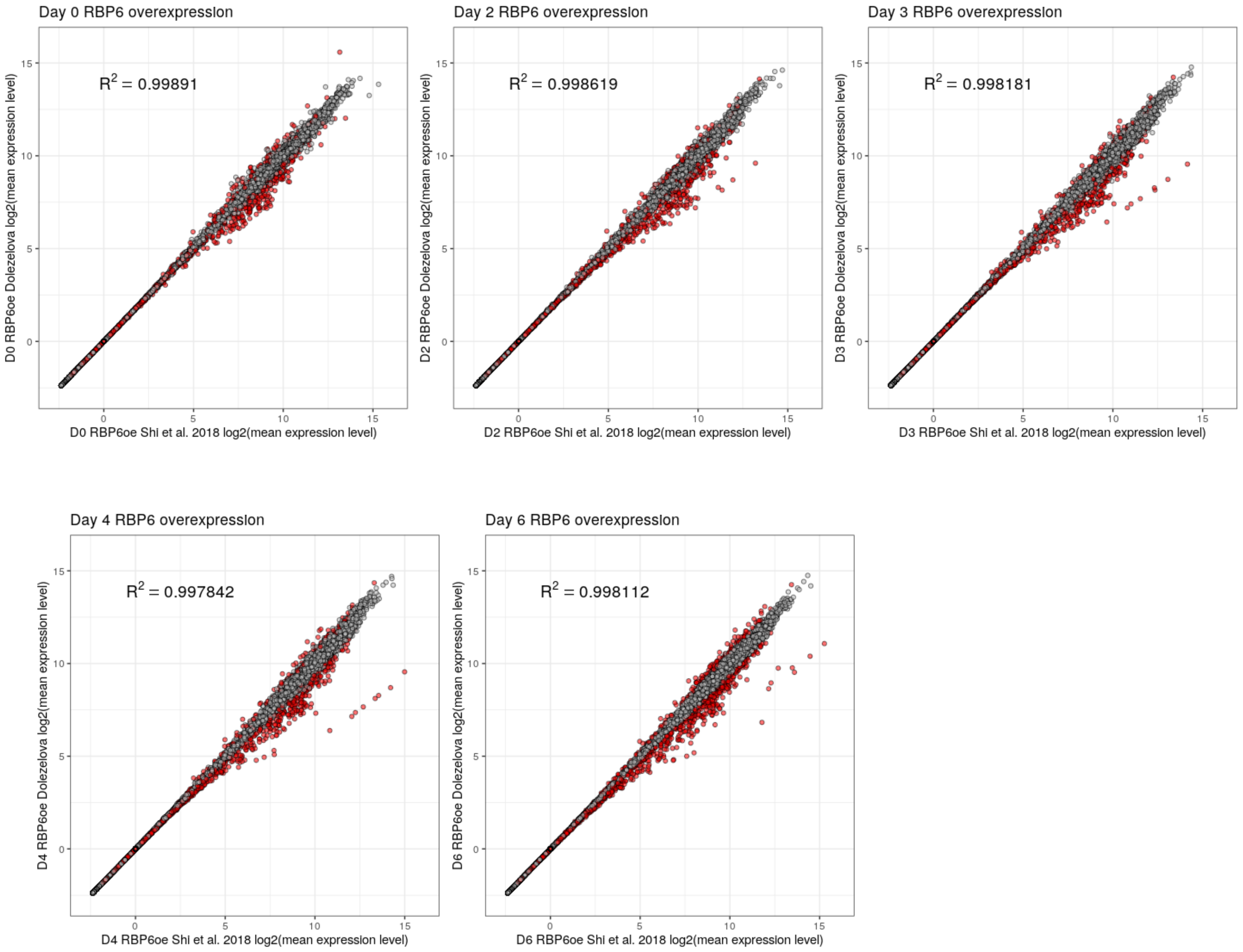
Day 0 RBP6 overexpression
R² = 0.99891
D0 RBP6oe Dolezelova log2(mean expression level)
D0 RBP6oe Shi et al. 2018 log2(mean expression level)
Day 2 RBP6 overexpression
R² = 0.998619
D2 RBP6oe Dolezelova log2(mean expression level)
D2 RBP6oe Shi et al. 2018 log2(mean expression level)
Day 3 RBP6 overexpression
R² = 0.998181
D3 RBP6oe Dolezelova log2(mean expression level)
D3 RBP6oe Shi et al. 2018 log2(mean expression level)
Day 4 RBP6 overexpression
R² = 0.997842
D4 RBP6oe Dolezelova log2(mean expression level)
D4 RBP6oe Shi et al. 2018 log2(mean expression level)
Day 6 RBP6 overexpression
R² = 0.998112
D6 RBP6oe Dolezelova log2(mean expression level)
D6 RBP6oe Shi et al. 2018 log2(mean expression level)
