# Supplementary material for: Cell-based and multi-omics profiling reveals dynamic metabolic repurposing of mitochondria to drive developmental progression of Trypanosoma brucei
Source: PLoS Biol. 2020 Jun 10;18(6):e3000741. doi: 10.1371/journal.pbio.3000741 (PMC7307792; doi:10.1371/journal.pbio.3000741)

Proteins involved in redox metabolism

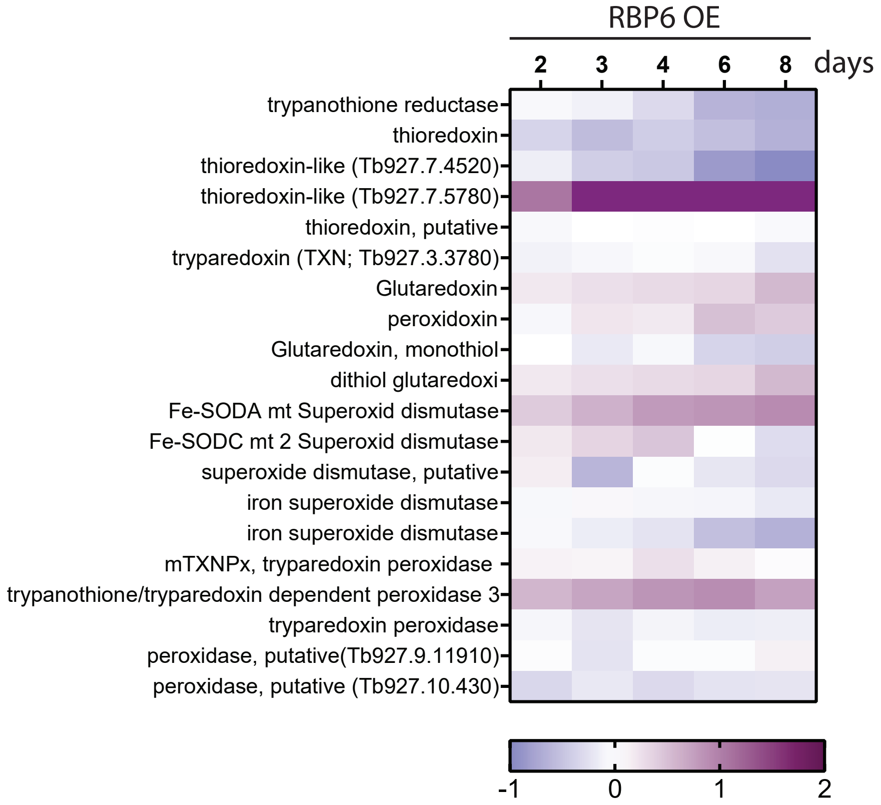

Mitochondrial carrier proteins (MCPs) and a putative citrate carrier

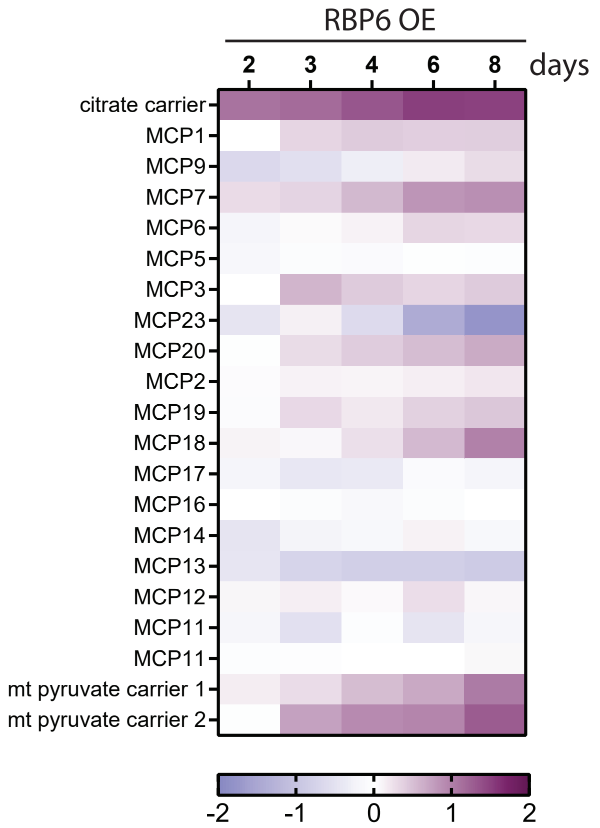

Supplement: S8 Fig — The color key differs for each map and is located below the heatmap. LFQ, label-free quantification; RBP6, RNA binding protein 6. (PDF) [file pbio.3000741.s008.pdf]
