# Supplementary material for: Cell-based and multi-omics profiling reveals dynamic metabolic repurposing of mitochondria to drive developmental progression of Trypanosoma brucei
Source: PLoS Biol. 2020 Jun 10;18(6):e3000741. doi: 10.1371/journal.pbio.3000741 (PMC7307792; doi:10.1371/journal.pbio.3000741)

S1_raw_images

Figure 1F

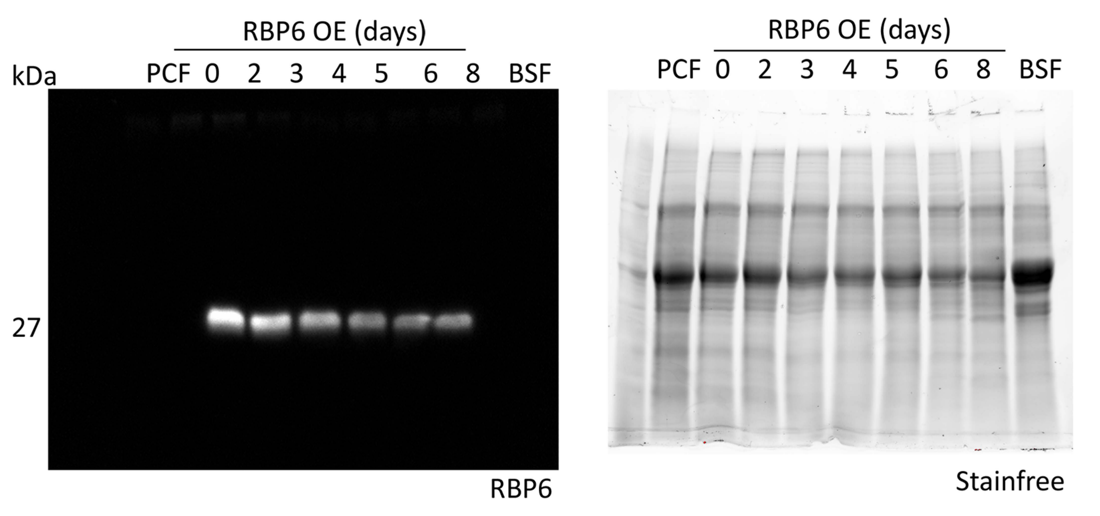

Figure 3B

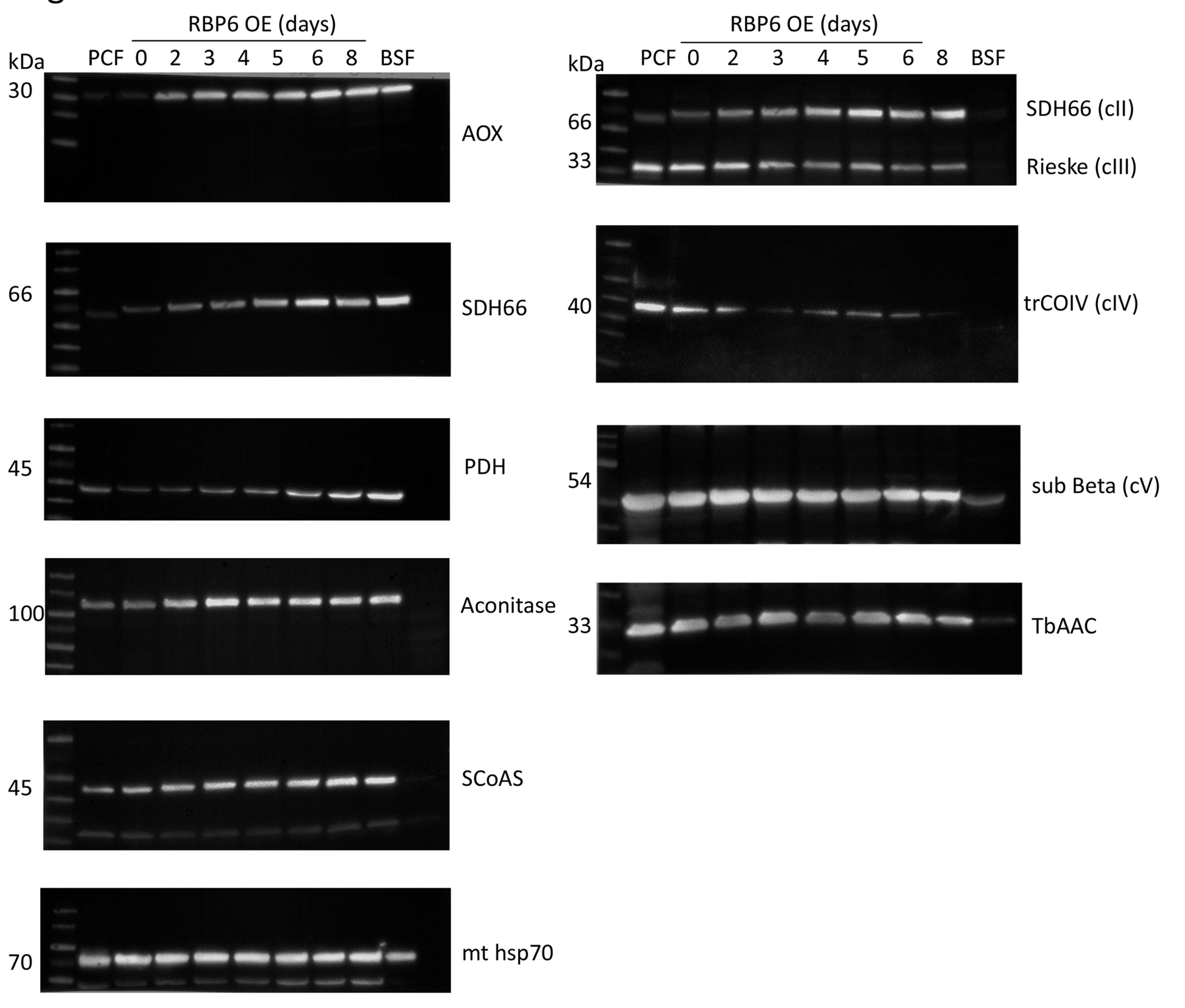

Figure 6

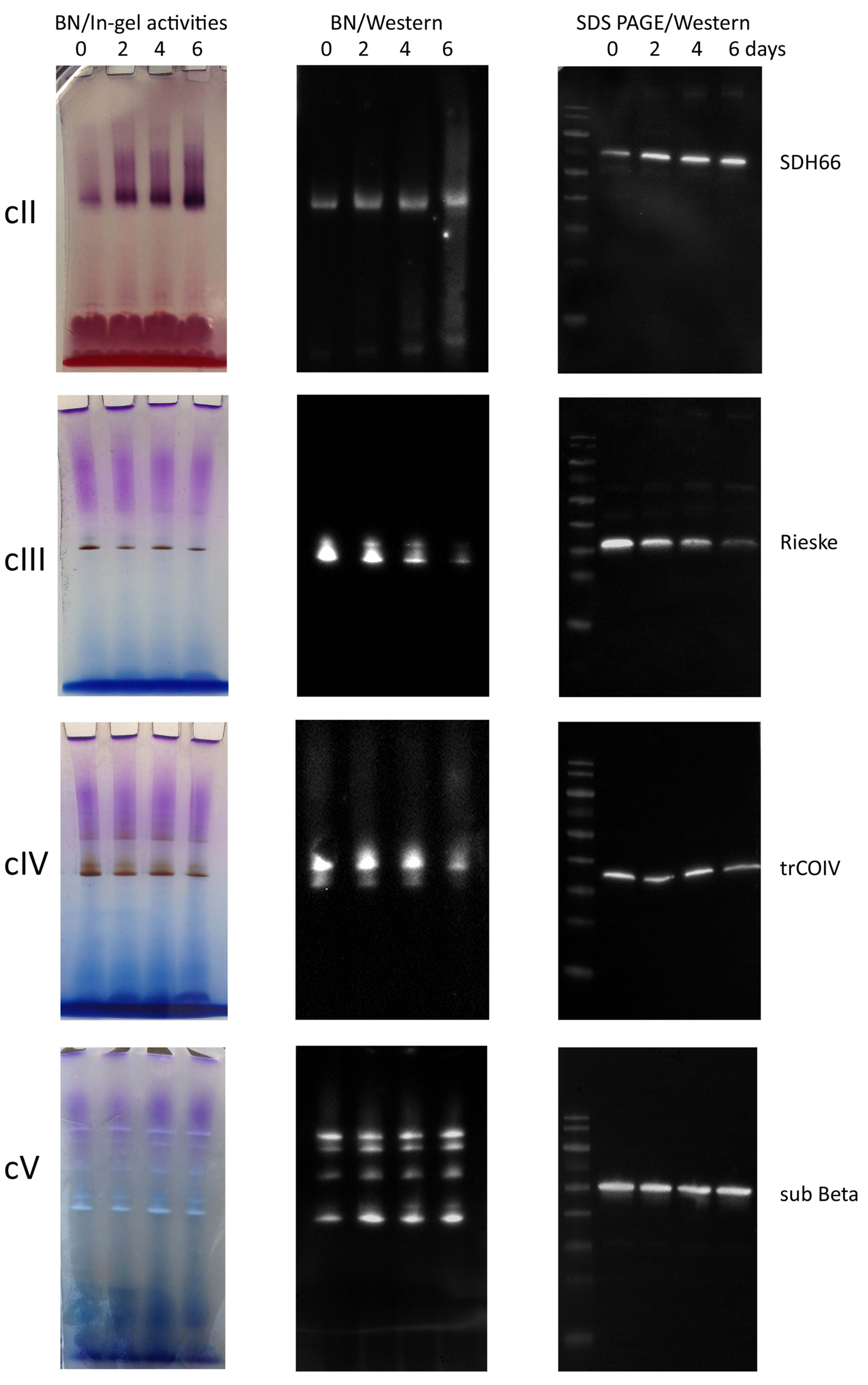

Figure 7C

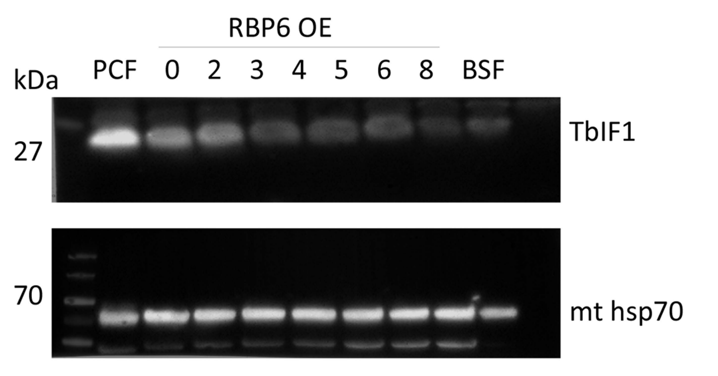

Figure 10C

Digitonine fractionation - catalase localization

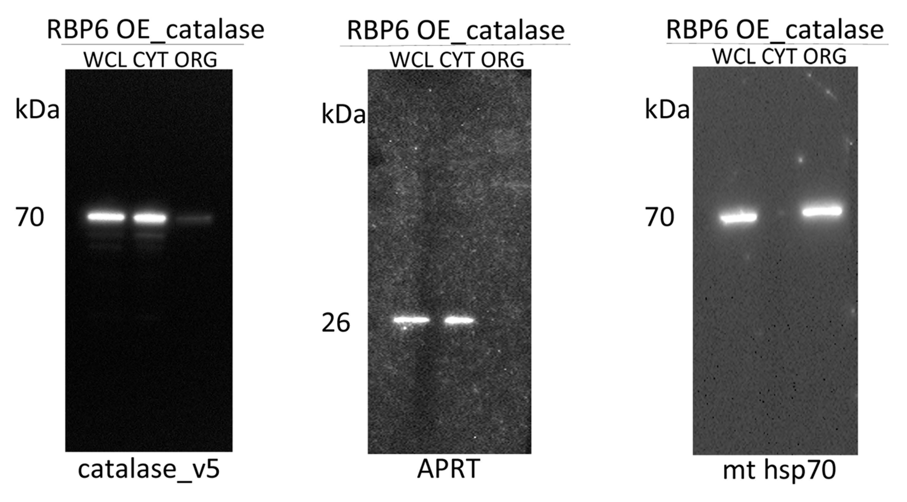

Figure 10E

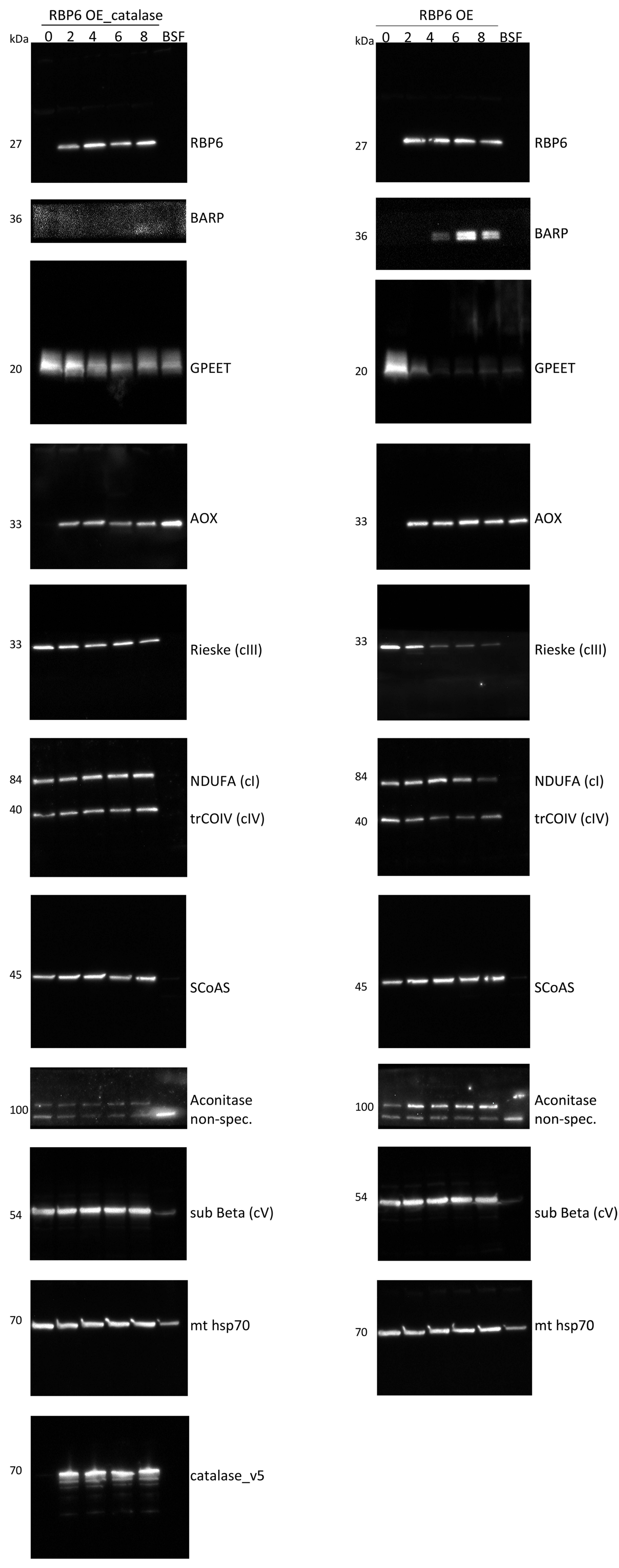

Supplement: S1 Raw Images — (PDF) [file pbio.3000741.s017.pdf]
