# Supplementary material for: Cell-based and multi-omics profiling reveals dynamic metabolic repurposing of mitochondria to drive developmental progression of Trypanosoma brucei
Source: PLoS Biol. 2020 Jun 10;18(6):e3000741. doi: 10.1371/journal.pbio.3000741 (PMC7307792; doi:10.1371/journal.pbio.3000741)
Supplement: Attachment — Submitted filename: Response to the reviewers.doc [file pbio.3000741.s018.doc]

*March 10^th^, 2020*

*Dear Dr. Richardson,*

*Thank you very much for your feedback on our manuscript. We also thank all the reviewers for taking time out of their busy schedule to provide us with fair criticism and recommendations that we believe have helped us to improve the quality of the manuscript. We agree about the possibility that, if accepted, our manuscript will be published as “Methods and Resources” article. Therefore, as requested, we highlighted the value of our datasets for further studies (lines 105-107 in the “track changes” version of the manuscript). These datasets have been already uploaded to the public depositories and will be made accessible immediately upon acceptance of the publication.*

*To satisfy the concerns raised by the reviewers we have included new data and thoroughly revised the manuscript. Therefore, we feel that the improved manuscript is now acceptable for publication in PLoS Biology.*

*Sincerely,*

*Alena Zíková*

**The point-by-point response to the referees comments**

**Academic Editor:**

Lastly, the Academic Editor notes that in lines 183-187 it is stated that “cluster 1 includes proteins that are being upregulated…in agreement with the transcriptomic data, it contains enzymes involved in energy metabolism….” but that this is not reflected in the figure.

*This has been corrected by specifying the cluster (cluster 4) in the transcriptomic data analysis that corresponds to cluster 1 in the proteomics analysis (lines 213-217).*

**Reviewer #1:**

Following uptake into the tsetse fly, African trypanosomes multiply as procyclic cells. After differentiation into epimastigote forms, infectious trypomastigotes are formed which are again transmitted to the mammalian host, closing the digenetic life cycle. The work submitted by Dolezelova et al. takes advantage of a previous finding that overexpression of RBP6, a specific RNA-binding protein, induces the differentiation process in vitro. The authors present thorough transcriptome, proteome and metabolome analyses with the aim to get a deeper insight in the changes occurring during the differentiation processes in the insect vector of Trypanosoma brucei. Most remarkably, they show that ectopic expression of catalase in the cytosol of procyclic parasites halts the in vitro-induced differentiation and suggest that hydrogen peroxide may act as signaling molecule.

The data presented are new and of significant interest especially for scientists working on the differentiation of African trypanosomes and related parasites. However, the manuscript requires a number of corrections and alterations.

**Major points:**

**1.** How long where the cells grown in the absence of glucose and presence of N-acetyl glucosamine before RBP6 overexpression was induced? Can the authors exclude that non-differentiating procyclic cells show similar alterations (protein expression profile, progressive proline and succinate consumption, ATP production etc.) when they are long-term cultured under these conditions? Non-induced cells cultured for six or eight days in the absence of glucose but presence of N-acetyl glucosamine should have been included for control.

*The cells were grown in the presence of glucose (SDM-79) during the selection period after the transfection of the RBP6^OE^ plasmid. Afterwards, the cells were adapted to the no-glucose media (SDM-80) with N-acetyl glucosamine for several weeks before stabilates were stored in liquid nitrogen. Therefore, RBP6 overexpression was always induced in cells that were already well adapted to these culture conditions. Under these conditions, the observed changes described in our manuscript can be contributed to the RBP6 overexpression and not due to a sudden change in the cultivating media. We have emphasized this fact in the text (Results: lines 117-118; Methodology: line 632).*

2. Figure 5, why is the O2-flux at day 0 in (A) resting cells as high as in (B) glycerol-3-phosphate-induced cells?

*Figure 5A shows routine respiration of living PCF using their endogenous substrates. The O_2_-flux per cell is steadily decreasing as cells are running out of the endogenous fuel. We thank the reviewer to point out that the way we presented our data was unfortunate and could have been misleading. We re-analyzed all experiments and Figure 5 has been simplified to show the ratio of KCN- and SHAM-sensitive respiration. In addition, we show the original data for representative experiments as Supplementary figure S5. From these figures, the rate of oxygen consumption and how the O_2_-flux per cell is affected by SHAM treatment can be simply deduced.*

3. Figure 6 and text, lines 265-269, please clarify the section and remove redundancies. The Western blots of complex IV at day 4 do not show any significant changes; lines 270-275, the description refers exclusively to day 2.

Please comment why at day 6, the activity of complex II is again as low as on day 0.

*We simplified this section and removed the redundancies (lines 296 -303). We also repeated the BN gels followed by activity staining for complex II twice independently. This data is more consistent and indicates that complex II is steadily upregulated during the programmed development, which is in agreement with the western blot analyses, transcriptomics and proteomics data (New Figure 6, complex II staining).*

4. Figure 8F, please explain the extremely high ADP/ATP ratio of 2, even on day 0. Usually, non-stressed cells have an ADP/ATP ratio of 0.1 to 0.2.

*We agree with the reviewer that ADP/ATP ratio detected in trypanosomes is very high compared to what is typically reported for mammalian cells. To provide further evidence that this measurment is accurate, we measured the ADP/ATP ratio again using a different ADP/ATP kit (abcam, ab65313). The manufacturer´s protocol was followed precisely and the results were similar to those obtained with the original kit (Sigma, MAK135-1KT). To streamline our data, we decided to plot relative ADP/ATP ratio (n=10) (new Figure 8F).*

5. Figure 10, C, please include the number of experiments and describe the Western blot in the legend, including the time point at which the samples were taken; D, please include the number of experiments; E and text, line 387, the statement about the relative AOX expression is not convincing as in the RBP6-OE-catalase sample (left blot), the AOX level in bloodstream cells is higher than in the RBP6-OE sample (right blot); and line 389, why are the aconitase levels in the catalase-expressing cells lower than in the cells not expressing catalase?; lines 392-397, the section is very speculative, especially " including signaling molecules" and should be modified; F, also in RBP6-OE cells without catalase (Fig 7E), the TMRE fluorescence increases upon induction; G and B, please use the same scale for the y-axis.

*1. We included the number of experiments and described the Western blot in the legend (lines 923-928)*

*2. We removed the statement about AOX expression levels*

*3. Western blots immunodetecting aconitase expression in RBP6^OE^_catalase and RBP6^OE^ cells were done independently at different times, which resulted in various exposure times optimized by the Chemidoc software. This may explain the slight differences in the band intensities between the RBP6^OE^ and RBP6^OE^_catalase noninduced cells. Otherwise, there is a clear trend of aconitase being up-regulated in the RBP6^OE^ cells in contrast to the unchanged expression in the RBP6^OE^_catalase cell line.*

*5. lines 392 – 397 were rephrased to minimize speculation, statement “including signaling molecules” was removed (lines 448 – 454)*

*5. We added “similarly to” to acknowledge that mitochondrial membrane potential as well as mitochondrial ROS production were increased in both cell lines (RBP6^OE^ and RBP6^OE^_catalase) (line 444)*

*6. We adjusted the scale for the y-axis in Figures B and G.*

6. Lines 45-46, should it read "aerobic cells"? Otherwise, the sentence is not clear.

*Corrected*

Line 225, the statement that glucose-6-phosphate isomerase functions only in the gluconeogenesis direction is not correct. Please define SBPase.¨

*Corrected. SBPase is defined in the text (line254-255) as well as in the Figure 1A legend (lines 833-837).*

Lines 296-298, the sentence starting with "Accordingly" is not clear.

*Sentence was shortened to emphasize the conclusion (330-333)*

Minor points:

1. For readers not specialized in the omics-field, the methodology should be described in more detail and the abbreviations (e.g. PCA; K-means, LFQ) defined in the text and/or figure legends.

*Corrected. All abbreviations are defined in the text and figure legends.*

The same abbreviation should be used throughout the manuscript (e.g. BF or BSF, PF or PCF; non-induced or uninduced; in Figure 3, glucose-6-phosphate isomerase is given as "P-6-glu isom" and in Figure 4 as Glu-6-P-ISO. In general, "glucose" should be abbreviated as "glc" not "glu", the three-letter code for glutamate). "Oxal" should be replaced by "oxaloacetate" etc.

*Corrected*

Please replace in line 36, "acted" by "appears to act" and in line 37, "exogenous" by "ectopic".

*Corrected.*

Line 183, please define the clusters 2, 5 and 6.

*Please see lines 210-213.*

Line 239, please replace "synthase" by "synthetase".

*Corrected based on definition that synthatases are enzymes that catalyze synthesis by using high energy nucleosides, while synthases catalyze synthesis without the need of NTPs.*

Line 313, please include (Fig 8A).

*Corrected.*

Line 315, AOX is not described in Fig 6; should it be Fig3B?

*We corrected the statement as it refers to the activity of complex II, succinate dehydrogenase, which is increased during the RBP6^OE^ (line 349-350)*

Lines 349-351, please describe more precisely which changes are consistent with the scheme in Fig 4?

*Please, see lines 389-392*

Line 355, the authors describe that the reduced form of trypanothione was not detected, what is with the oxidized form?

*The oxidized form of trypanothione was not found to be significantly altered across the time course of RBP6OE (lines 404-407).*

Line 416, please modify the sentence. It should read "we detected lower levels of proline and glutamate which may suggest a higher consumption".

*Corrected.*

Line 434, replace "synthetase" by "synthase".

*Corrected*

Line 482-484, to get a deeper insight in the subcellular origin of hydrogen peroxide, catalase could be expressed in the mitochondrial matrix.

*Yes, indeed, this experiment is in progress in our laboratory.*

Figure 2D, it should read "Protein phosphorylation"

*Corrected*

Figure 3A, "day" and "log 2-fold", respectively, should be included in front of the numbers.

*Corrected*

Figure 4, the use of different colors for the upregulated reactions would facilitate reading. All abbreviations should be defined in the legend. Pyruvate is given twice in grey boxes, ones without, please clarify.

*The pyruvate was highlighted twice in grey boxes to emphasize that it is the same molecule, but for clarity we removed the boxes. The figure legend was expanded to include all enzyme and substrate abbreviations.*

Figure 5C, what is the meaning of s.d. for only two values?

*We agree with the reviewer and we repeated the assay to include more data points.*

Figure 7 legend, include after "cells" "that", see also comment for Figure 5

*Corrected. We also repeated the assay and added more independent measurements.*

The supplementary figures S1-S5 were missing in the manuscript provided and thus were not evaluated.

*Probably an unfortunate position, but the supplementary figure S1-S5 were provided at the end of the submitted pdf behind the Figures.*

---------------

**Reviewer #2:**

Trypanosoma brucei lives in two hosts, mammals and tsetse flies, and adapts its metabolism to its different environments. Until recently, the majority of life-cycle stages from tsetse could not be cultured. In this study the authors exploit a system first described by Kolev et al., over-expression of the RNA-binding protein RBP6. This drives differentiation in culture from the procyclic (fly midgut) form, via a series of intermediate life-cycle stages, to the metacyclic form which is infectious for mammals. In contrast to Kolev et al., the authors use a glucose-free medium, which appears to make differentiation more efficient. This study provides time courses of the transcriptome, proteome and metabolome, with a focus on the mitochondrion which changes during development. Interestingly, reactive oxygen species seem to play a role in differentiation as expression of catalase (an enzyme not normally present in trypanosomes) blocks life-cycle progression in cultures.

I appreciate this work as it contains a wealth of data, but I would have liked to have seen it analyzed more extensively. The authors focus on the metabolic capability and remodeling of the mitochondrion, an organelle that is present in almost all eukaryotes. Nevertheless, for the following reasons, I am in two minds about whether it fits the scope of PLoS Biology or if the data would be more appropriate for another PLoS journal (e.g. PLoS Pathogens).

*We are glad that the reviewer appreciates our work and we would like to thank him/her for the valuable comments. We agree that the data presented in this work represents a wealth of information that could have been analyzed on a more global scale. However, the main long-term interests of the lab revolve around how T. brucei contains a unique single mitchondria that is drastically remodeled metabolically and structurally as it alternates between two diverse hosts during its complex life cycle. While the complete -omics data sets will be available for other research groups in the community to download and extract information about their favorite aspects of Trypanosoma biology, we believe that a mito-centric narrative that includes more specific bioenergetic functional outcomes that complement the omics-data allows us to delve deeper into the role of the mitochondrion during in vitro differentiation. In our opinion, this is the strength of the manuscript as it communicates a compelling reason why this genetically tractable protist can serve as a model organism to study how mitochondria can transmit signals within the cell to adapt to various environments.*

*rests in mitochondria biogenesis, metabolism and bioenergetics given the striking plasticity of this organelle to quickly adapt to environmental changes and to serve not only to synthesize ATP and metabolites but as well to communicate with rest of the cells via various signals. We performed our –omics analyses to carefully look at the mitochondria behavior during the in vitro differentiation and to detect possible signaling molecules which can be further analyzed. The scientists are welcome to download our datasets and extract information for their favorite pathways. In our opinion, this is the strength of this manuscript as it describes in details interesting biological phenomena of mitochondria repurposing as well it presents multi-omics datasets which will be of high interest to the community interested in other exciting aspects of Trypanosoma biology.*

1. The work sheds most light on differentiation and could identify new stages or intermediate stages in the life cycle. It might then be easier to attribute metabolic pathways to specific stages of the life cycle.

*The RBP6OE system is a powerful tool to get deeper insights into the developmental progression of T. brucei. However, it has its limitations since the induced culture is a mix of various life cycle stages; defined ones (early and late procyclics, epimastigotes, metacyclics) or their intermediates (e.g. procyclin-positive epimastigotes). We agree that it would be very informative to attribute certain metabolic pathways to specific stages, but to do so, one would have to first enrich for these specific stages. Unfortunately, our -omics datasets are only able to assess global changes that describe various trends occurring during differentiation. Therefore, it is our opinion that our datasets do not represent a great tool to identify specific new life cycle stages. One way to achieve this would be to find specific markers for different sub-populations. Presumably, the UTR’s of a regulated marker could be fused to a fluorescent protein that is then used to sort the parasites. Then any analysis of these enriched cell populations would be highly informative. Such an approach is currently being implemented in our lab. Otherwise, use of single cell sequencing may help to identify new intermediate stages.*

2. There are nuances in the system that are difficult for non-parasitologists to appreciate. The differentiating cultures consist of mixtures of cells, some of which do not usually coexist in one tissue. The data thus reflect these mixtures overlaid upon each other. This differs from a previous study by Cristiano et al., in which in vitro-derived metacyclics were purified prior to transcriptomic and proteomic analyses. Incidentally, their data strongly suggested that metacyclic forms and bloodstream forms are metabolically similar. Importantly, by day 8 in the current study, there is a considerable proportion of "procyclic cells" (more about this below). This means that the cells in culture are either dedifferentiating and/or that there remaining epimastigotes are non-dividing and are being overgrown by procyclics. This may also explain why the clusters of co-expressed transcripts almost all change their trend on day 8.

*We agree with the reviewer that since we used mixed cell populations and analyzed trends over time, our analyses differ from the previous study by Cristiano et al, in which glucose-grown metacyclics cells were enriched on zirconium beads prior to the –omics analyses. However, we are a little unsure of the reviewer´s point here as there were clear differences between the metacyclics and bloodstream forms. The Cristiano et al. concluded that metacyclic cells have a largely bloodstream-like transcriptome, but metabolically they exhibit features of both PCF and BSF cells. Our transcriptomic data show that day 8 is most similar to the pure metacyclics (Figure S2), but it is true that the day 8 culture contains a considerable proportion of procyclic cells. We have extended the description of the RBP6OE induced culture to increase the awareness that the culture contained mix cell populations of various life cycle stages (lines 137-143).*

3. Although the complex life cycle is clearly described, it is not sufficiently clear how different stages were categorized. For example, in Figure 1A, panel day 2 epimastigotes: only one cell has the classic anteronuclear kinetoplast. One cell has a procyclic configuration. The other two might be transitioning as the kinetoplast is very close to or over the nucleus.

Intriguingly, if the majority of cells on day 2 are epimastigotes, they are not expressing BARP proteins (see Figure 10E). While it is known what salivary gland epimastigotes express BARPs, it is not known if they are expressed by epimastigotes in the foregut. Might there be two populations of epimastigotes in these cultures and are their mitochondria and metabolism different? The authors may be able to extract some more information from their data sets.

*We would like to thank the reviewer for pointing out the complexity of the RBP6^OE^ differentiating cells in vitro. We appreciate his/her insightful comments but we are not in a position to comment further at this point. Future work using single cell RNA sequencing might indeed point towards mixed populations and we look forward to having an opportunity to doing this.*

*To make it clear how the different stages were categorized, we extended the results section (please, see lines 129-136). We also added a graph depicting distances between kDNA and nucleus for 50 cells for each tested day to visually express the emergence of an epimastigote population (new Figure 1B). It is true that the epimastigote population was quite heterogeneous with regards to their surface marker molecules. At day 2, most of the cells were still expressing procyclin, while later during the RBP6^OE^ expression the epimastigote cells expressed BARP on their surface. To address this complexity, we included two new figures showing immunofluorescene analysis of RBP6OE cells at day 2 and 6 post-induction using anti-procyclin and anti-BAPR antibodies (new Figure 1C and D). We also quantified the procyclin-positive and BARP-positive populations by FACS and compared them to RBP6^OE^_catalase cells (as also requested in comment 6). The graphs are now shown as Figure 10F.*

*Our data suggest that there are at least two population of “epimastigote” cells, one expressing procyclin (day 2-4) and one expressing BARP (day 4-6). This is now acknowledged in the expanded result section (lines 158-171). We also compared the transcriptomics data from these data points, but it was not possible to extract any specific traits. A method that would enrich for the specific cell populations would allow such comparison.*

*The metacyclic cells were scored based on their homology, but also on their ability to uptake fluorescently-labeled dextran. We added a representative figure of RBP6^OE^ day 8 culture treated with dextran (new Figure 1A, right panel)*

4. The procyclic forms on day 8 do not reflect the natural life cycle. They don't seem to express GPEET procyclin protein or mRNA. What about the other procyclin marker, EP? This information could be extracted from the RNA-seq data. If they are procyclic forms, it should be made very clear that this is a culture artefact. Alternatively, it might be better to remove most of the day 8 data.

*We agree with the reviewer that we should have addressed the expression of surface marker molecules in more detail to better explain the mixed character of the culture upon RBP6 overexpression. We have included the expression profile for all three EP transcripts in Figure 2B. It is obvious that expression of EPs, the marker for late procyclics, is increased on day 2 of RBP6^OE^ and that EP transcripts were detected throughout the experiment. This is now acknowledged in the text (170-171). We would like to keep the day 8 data since this data point was the closest one to the pure metacyclic cells (Supplementary figure S2) and thus we would lose valuable data.*

5. RNA-seq data (Table S1). The gene IDs are from the 2018 version of T. brucei 427. Although this makes sense, because this cell line is a derivative of 427, it makes it harder to browse the data when it comes to differentially expressed genes. Would it be possible to include the orthologs in T. brucei 927 and gene names in all of the tables?

*Tb927 orthologues were added to Table S1.*

6. The role of ROS and the effect of ectopic catalase are intriguing. It was shown recently that trypanosomes expressing catalase were less successful at establishing midgut infections. Based on the data shown here (presence of GPEET absence of BARP), it looks like the block in differentiation occurs much earlier than the transition from epimastigotes to metacyclic forms. Analyzing the cells in these cultures by immunofluorescence or flow cytometry, in addition to morphology, would be informative. Are the majority procyclic forms (expressing GPEET) or is this a sub-population?

*We agree with the reviewer that the developmental progression of RBP6OE_catalase cells is blocked before the transition of mature (BARP positive) epimastigotes to metacyclics. As suggested by the reviewer we performed flow cytometry analysis which shows that RBP6^OE^_catalase cells stay procyclin-positive, BARP-negative over the period of 8 days in contrast to RBP6^OE^ cells in which this procyclin marker is down-regulated at day 4 after the induction and a BARP-positive subpopulation appears (new Figure 10F). Detailed analysis of the RBP6^OE^_catalase cell line, as well as an investigation of the molecular mechanism behind the effect of catalase expression in RBP6^OE^ cells is still progressing in the laboratory*.

7. The metabolome is hardly discussed apart from metabolites that change in concentration over the course of the experiment. Can more information be extracted? Are there indications of unusual pathways or missing pathways?

*Unfortunately we were not able to identify any major pertubances (e.g. unusual pathway or missing pathways) during the RBP6^OE^, but we tried to expand the metabolomics description (see lines 377, 386-390, 398-404, 406-408).*

Minor points:

8. "Morphotype" is not a word that is normally applied to trypanosomes. Should this be life-cycle stage? Monoformic (monomorphic?). This refers to bloodstream forms that have been syringe-passaged until slender forms lose the capacity to differentiate to stumpy forms.

*Here the word morphotype is used to replace commonly used “life cycle stage”. It has been used in the literature by various scientists for example Prof. Engstler (Krueger et al., 2018), Prof. Rotureau (Rotureau et al., 2012), Prof. Carrington (Sharma et al., 2009). This word does not refer to cells which are monomorphic (i.e. cells which lost ability to differentiate to stumpy forms in vivo). We respectfully disagree with the reviewer and we would like to keep this word as an alternative to life cycle stage to keep the text word-rich.*

9. The word "and" is missing from line 119. It should be … typically smaller than epimastigotes and PCF, and are ….

*Corrected.*

---------------

**Reviewer #3:**

In this manuscript Doleželová et al took advantage of a previously established in vitro differentiation system based on the over-expression of the RNA binding protein 6 (RBP6) to follow changes at the RNA, protein and metabolite levels during the development of Trypanosoma brucei procyclics to infectious metacyclics. Although this development takes place in the tseste fly vector, investigations in the fly are not feasible due to difficulties in acquiring enough parasites, especially for proteomic and metabolomic studies. Thus, the RBP6 over-expression system provides an ideal model system. The authors performed a careful multi-omics study and provide convincing evidence for redirection of electron flow from the cytochrome mediated pathway to an alternative oxidase. This is a rich set of data that will be very valuable to the community. Most importantly, they not only provide insights into the mechanisms of the parasite´s mitochondrial rewiring, but also strengthen the

emerging concept that mitochondria act as signaling organelles through the release of reactive oxygen species to drive cellular differentiation.

There are only a few minor points that will need attention.

1. A recent publication (MBP 224, 50-56, 2018) describes an RNA-seq analysis of the time course of RBP6 induction. Although the data presented here are more detailed due to the higher efficiency of differentiation, a comparison of the results is warranted.

*We compared our RNAseq data with the data published in Shi et al., 2018. The results of this analysis are now presented as new Supplementary Figure S2B as well as Supplementary Table S3. The respective results were expanded to reflect this analysis (lines 186-192).*

2. The sentence starting on line 333 needs a reference.

*Reference added.*

3. Line 496: "Even though RBP6OE is not the physiological route to differentiation of the midgut PCF trypomastigote, " What do the authors mean? Do they have evidence for the physiological route to differentiation?

*“…not the physiological route…” was replaced with “ is genetically-induced and thus artificial route…” line 556*
