# Supplementary material for: Cell-based and multi-omics profiling reveals dynamic metabolic repurposing of mitochondria to drive developmental progression of Trypanosoma brucei
Source: PLoS Biol. 2020 Jun 10;18(6):e3000741. doi: 10.1371/journal.pbio.3000741 (PMC7307792; doi:10.1371/journal.pbio.3000741)
Supplement: Attachment — Submitted filename: Rebuttal letter_fin_R3.doc [file pbio.3000741.s019.doc]

May 13th, 2020

Dear PLOS Biology editors,

Thank you very much for your feedback on our revised Methods and Resources article entitled "Cell-based and multi-omics profiling reveals dynamic metabolic repurposing of mitochondria to drive developmental progression of *Trypanosoma brucei*". We also thank the reviewers for taking time out to review our revised manuscript. The reviewer had two additional minor inquiries that we have addressed in the submitted version of this manuscript. We also updated the submission form and the manuscript based on PLOS formatting requests and we followed the PLOS data policy and included S1 Data file and S1 raw image file. The –omics data have been deposited in publicly available repositories.

We hope that this version is now acceptable and look forward to hearing from you.

Sincerely,

Alena Zíková

***The point-by-point response to the referees comments***

***Points raised by reviewer 1:***

1. Most of the major points have been dealt with, including the issue of pre-adapting the cells to glucose-free medium. I am not sure if the reviewer would have been satisfied by the responses to his/her points 2 and 4, where the data are now depicted as ratios. These do not really address the questions about absolute values. For point 4, the authors used two independent kits to determine ADP/ATP levels, which adds credence to their findings, but they avoid mentioning that the levels of ADP are unusually high by using a "normalised" ratio of 1.

**We kept the normalized ratio of 1 in Fig 8F, but we clearly stated in its legend, that the measured values were unusually high for unknown reasons. The determined values are also listed in S1 Data file. Please see lines 483 - 486.**

***Points that I* (the reviewer) *raised.***

2.Again, most have been dealt with satisfactorily. There is one discrepancy, however. In the Western blot in Figure 10E, BARP is not detectable in cells expressing catalase, but is detected in Figure 10F (flow cytometery) in about 10% of cells. This may be a sensitivity issue, but it should be addressed.

**This discrepancy can be explained by the differences in how the samples were treated. For the Western blot analysis, the *T. brucei* RBP6^OE^ cells were harvested and immediately lysed in SDS PAGE buffer (Fig 10F), while for the FACS analysis the RBP6^OE^ cells were pre-treated with 5 mM bathophenanthroline disulphonic acid (BPS), a metalloprotease inhibitor to stabilize surface proteins such BARP as suggested by the literature (Urwyler et al., 2007). This treatment step is clearly stated in the relevant Material and Methods sections as well as in the Fig 10F legend. Lines 561 – 563, 893 -894, 909.**

3. Please check the revised version for typos, etc. There are several places where spaces are missing between words, letters were transposed or singular and plural didn't match.

Media should be medium (if it is only one). "Mixture" might be more appropriate than "blend" to describe the cultures.

**We are sorry for the overlooked typos. The whole text was run through Grammarly software and proof-read several times to minimize typos.**
